# Origin of static magnetic field induced quality improvement in sea bass (*Lateolabrax japonicus*) during cold storage: Microbial growth inhibition and protein structure stabilization

**DOI:** 10.3389/fnut.2022.1066964

**Published:** 2022-11-16

**Authors:** Li Tong, Haiqing Tang, Jingyi Chen, Shangyuan Sang, Ruiping Liang, Zhepeng Zhang, Changrong Ou

**Affiliations:** ^1^College of Food and Pharmaceutical Sciences, Ningbo University, Ningbo, China; ^2^Faculty of Food Science, Zhejiang Pharmaceutical University, Ningbo, China; ^3^Key Laboratory of Animal Protein Food Deep Processing Technology of Zhejiang Province, Ningbo University, Ningbo, China

**Keywords:** sea bass, static magnetic field, myofibrillar protein, total viable counts, cold storage, protein structure

## Abstract

To explore the potential application of static magnetic field (SMF) treatment in marine fish preservation, the sea bass (*Lateolabrax japonicus*) was exposed to SMF (5 mT) and its quality changes during cold storage were evaluated by total viable counts, water holding capacity, pH, color, and textural properties. Characteristics of the protein in the presence of SMF were investigated by measuring total sulfhydryl (SH) content, Ca^2+^-ATPase activity, secondary structure, and muscle microstructure. SMF treatment exhibited positive effects on fish quality, showing favorable performance on the most quality indicators, especially a significant reduction in the Microbial Counts. Furthermore, higher total SH content and Ca^2+^-ATPase activity were observed in SMF-treated samples, demonstrating that the oxidation and denaturation of myofibrillar protein (MP) were delayed due to SMF treatment. The transformation of α-helix to random coil was prevented in SMF-treated samples, indicating that the secondary structure of MP was stabilized by SMF treatment. The above changes in protein structures were accompanied by changes in muscle microstructure. More intact and compact structures were observed in SMF-treated samples, characterized by well-defined boundaries between myofibers. Therefore, our findings suggest that under the conditions of this article, SMF treatment could maintain the quality of fish mainly by inhibiting the growth of microorganisms and enhancing the stability of protein structures, and could be a promising auxiliary technology for preservation of aquatic products.

## Introduction

Fish and fish products play an increasingly important role in human diet due to their delicious taste, rich nutrients, and widely recognized health benefits (1). However, the presence of relatively large amounts of protein and polyunsaturated fatty acids in fish, although with recognized benefits for health, is a significant obstacle for fish preservation due to their instability in the presence of external spoilage microorganisms and endogenous proteolytic enzymes (2, 3). Preservation of aquatic products has become a quite challenging and urgent issue. It is well known that low-temperature storage has been extensively used in aquatic products to extend shelf life and ensure quality. Thereinto, frozen and cold storage are considered to be the most common and effective methods to prolong the freshness lifetime of food by controlling the growth of pathogenic and spoilage microorganisms and slowing down the activity of endogenous enzymes (4). During the freezing process and frozen storage, the free water in fish muscle would undergo a liquid-solid transition to form ice crystals, which would severely damage the cell structure and thus lead to the quality deterioration of fish (5, 6). Consumers express personalized and diversified desires for organoleptic and nutritional quality in fish (7). Fish with compromised quality after frozen storage could not meet consumer demand for premium quality fish. Unlike the subzero temperature of frozen storage, temperatures in cold storage rooms range from 0 to 4°C. In this temperature zone, the water in food often exists in a liquid state. However, the relatively high storage temperature corresponds to a shorter shelf life, depending upon the type of products (8). Thus, researches on extending the fresh-keeping period of non-frozen aquatic products have attracted increasing attention.

For the above purpose, many emerging approaches, alone or combined, have been developed and applied, including nanoparticles, cold plasma, active packaging and so on (2, 9, 10). According to preservation mechanisms, these available technologies could be classified into chemical, biological, and physical approaches. Regrettably, while chemical and biological treatments are effective measures to inactivate spoilage bacteria and inhibit enzyme activity in aquatic products, consumers are concerned about the flavor changes and foodborne illnesses that these treatments might bring (11). As a result, many non-thermal physical methods have been applied to the preservation of fish, such as high-pressure processing (12), pulsed electric field (13), and cold plasma (9). However, there are still some unresolved issues with the above technologies, such as the difficulty of high-pressure processing for continuous production, the unsuitability of pulsed electric field for complex food systems (14), and the significant changes in food sensory quality after cold plasma treatment (15).

Static magnetic field (SMF) treatment is a novel physical preservation method characterized by high penetration depth and no reagent residue (16, 17). SMF treatment was initially proposed for the medical cryopreservation of biological organs and tissues and the germination of seeds (18, 19), and introduced into food preservation in recent years (20, 21). The major components in food are diamagnetic substances, of which functional properties were influenced by the external magnetic field. For instance, the SMF treatment has proven to strengthen intramolecular-hydrogen bonds and depress self-diffusion coefficient, thereby increasing the water activity and promoting a reduction in drip loss and a delay in tissue softening (22).

To date, few published researches mainly concentrated on the field of SMF-assisted frozen storage (23, 24). Nevertheless, given the above-mentioned disadvantages of frozen storage, the combined treatment of SMF and cold storage appears to be a more promising approach for maintaining freshness and obtaining better sensory quality in fish. According to the study of Lin et al. (25), SMF treatment (7.98–8.15 mT) combined with supercooling storage extended the shelf life of beef without adverse effects on other quality characteristics. Moreover, Bajpai et al. (26) found that SMF treatment (100 mT) effectively inhibited the growth of *Staphylococcus epidermidis* (gram-positive bacteria) and *Escherichia coli* (gram-negative bacteria) by disrupting the integrity of bacterial cell membranes. Interestingly, some studies have also found that SMF treatment had no positive effect on the preservation of food, and SMF treatment even promoted the growth of certain microorganisms, such as *Pseudomonas aeruginosa* (27). Hence results published in the literature are apparently contradictory. Further studies should find some clear evidence that SMF treatment is beneficial for food preservation and elucidate the underlying mechanism of action.

Magnetic fields (MFs) could be classified as time-varying magnetic field or SMF according to time interval (28). The time-varying magnetic field, which is associated with changes in electrical current, has strict requirements on voltage and current, so applying it as an auxiliary technology may significantly increase equipment costs and operating conditions. In practice, the heat generated by current coils of time-varying magnetic field could not be ignored (29). The SMF produced by a steady current in the surrounding space has constant magnetic field intensity and direction. Since the voltage and current requirements of SMF treatment could be easily met, it can be more suitable to be an adjunct on existing cryogenic storage systems such as refrigerators. In addition, considering the biological effects caused by SMF, it is accordingly classified as weak (<1 mT), moderate (1 mT–1 T), strong (1–5 T), and ultra-strong (>5 T). Zhao et al. (30) indicated that strong magnetic fields may damage the properties of cells and tissues and should not be applied to preserve fresh food. Moreover, a very weak magnetic field was also ineffective for food preservation. As reported by Zhu et al. (31), the effects of SMF treatment on retarding the deterioration of shrimp (*Litopenaeus vannamei*) could already be observed at an intensity of 5 mT. Hence in this study we focus on the effects of exposure to SMF of 5 mT on fish quality during cold storage, considering the reasonable energy requirements and future commercial applications.

The present study aims to investigate the effects of SMF treatment on the quality of sea bass during cold storage by evaluating microbial quality, physicochemical properties, and protein structure, and provide theoretical support for the further application of SMF treatment in the aquatic product industry.

## Materials and methods

### Sample preparation

A total number of 14 live sea bass (weight 600 ± 35 g, length 31.5 ± 3.5 cm) were purchased from a local aquatic market (Ningbo, Zhejiang, China) in October, and transported to the laboratory in a box filled with water containing dissolved oxygen. Once arrival, the live sea bass was killed immediately by a physical blow to the head with a wooden hammer and then peeled, headed, and gutted. The dorsal muscle of sea bass was cut into fillets, which were individually packaged with polyethylene bags, and finally the fillets were randomly divided into two equal batches. The fish fillet subjected to SMF treatment (5 ± 0.1 mT) in a magnetic field-assisted refrigerator (MFI-Fm-x1, INDUC Scientific Co., Ltd., Wuxi, Jiangsu, China), and the temperature was set at 4 ± 0.1°C. The magnetic field-assisted refrigerator consisted of a magnetic field generator, a biological sample chamber, and a temperature control and monitoring system. A power supply and a pair of Helmholtz coils (80 cm × 80 cm square; 400 turns) constituted the magnetic field generator, which can produce a uniform magnetic field of 0–5 mT at an excitation current of 0–8 A. The magnetic field uniformity was 99%, verified by Maxwell Software simulation. The biological sample chamber had a volume of 50 L, and the temperature ranged from −20 ± 0.1 to 40 ± 0.1°C. The control treatment conditions were consistent with those of the experimental group. The control treatment condition was consistent with the experimental group except for exposure to SMF. The fillets were taken each day during storage for subsequent analyses.

### Determination of total viable counts

Total viable counts (TVC) of the sea bass fillet were performed by the method of Hernandez et al. (32). An aliquot of 10 g minced sea bass fillet was placed in a sterile homogeneous bag with 90 ml of 0.9% normal saline and homogenized with a Masticator paddle blender (basic panoramic, IUL S.A., Spain). The homogenate was diluted with 0.9% normal saline and inoculated on the medium. TVC were determined by the pouring method in plate count agar (PCA) mediums. The PCA medium was purchased from Hangzhou Microbial Reagent Co., Ltd. (Hangzhou, China). The inoculated plates were incubated at 30 ± 0.5°C for 72 h. Results were recorded as log_10_ CFU (colony forming units)/g.

### Determination of water holding capacity

Water holding capacity (WHC) was expressed as a percentage of weight loss of the initial sea bass fillet. Approximately 5 g of non-minced fillet was wrapped with filter paper, and centrifuged at 4,000 × *g* for 15 min at 4°C. Then, the water was poured out of the centrifuge tube and the remaining fillet was weighed again. The measurement was carried out in triplicate. The WHC was calculated by the Equation 1:


(1) $$\text{WHC}\left(\%\right)=\left[1-\frac{\left(m_{1}-m_{2}\right)}{m_{1}}\right]\times 100\%$$

where *m*_1_ is the weight of the sea bass fillet before centrifugation, and *m*_2_ is the weight of the fillet after centrifugation.

### Determination of pH

An aliquot of 5 g minced sea bass fillet was homogenized with a homogenizer (NANOJ H10, ATS Engineering Inc., Germany) in 50 ml of 0.1 M KCl (pH = 7.0). The pH of the homogenate was determined by a pH meter (PHS-2F, Shanghai INESA Scientific Instrument CO., Ltd, Shanghai, China).

### Determination of color

The color of the fish fillet was measured by a colorimeter (NR110, Shenzhen 3nh Technology CO., LTD., Shenzhen, China) according to the method proposed by Chmiel et al. (33). Each fillet (50 mm × 20 mm × 10 mm) was measured at three typical positions (anterior, middle, and posterior) with recording L* (lightness), a* (redness/greenness), and b* (yellowness/blueness). All measurements were analyzed in five replicates, from which an average was calculated. The absolute color difference (ΔE) was calculated by the Equation 2:


(2) $$\Delta \,E=\sqrt{{\left(L_{\text{i}}^{*}-L_{0}^{*}\right)}^{2}+{\left(a_{\text{i}}^{*}-a_{0}^{*}\right)}^{2}+{\left(b_{\text{i}}^{*}-b_{0}^{*}\right)}^{2}}$$

where $L_{0}^{{}^{*}}$, $a_{0}^{{}^{*}}$, and $b_{0}^{{}^{*}}$ represent the color parameters of the fresh sea bass fillet. $L_{\text{i}}^{{}^{*}}$, $a_{\text{i}}^{{}^{*}}$, and $b_{0}^{{}^{*}}$ represented the color parameters of the fillet during storage.

### Texture profile analysis

The texture profile analysis (TPA) was measured by a texture analyzer (TA-XT plus, Stable Micro Systems, Surrey, UK). The fish fillet was cut into cubes (30 mm × 20 mm × 10 mm) and equilibrated at room temperature (25°C), then compressed to 40% of its initial thickness using a spherical probe having a one-inch diameter (P/1S), with a trigger force of 5 g and a test speed of 1 mm/s. Texture performances were expressed as hardness, springiness, cohesiveness, and chewiness. The hardness was defined as the maximum positive force of the first compression, expressed in grams. The cohesiveness was defined as the ratio of the positive area of the second compression to the positive area of the first compression. The springiness was defined as the ratio of the height detecting of the second compression to the first compression distance. The chewiness was defined as the product of hardness × cohesiveness × springiness. At least five replicates were performed for each treatment, once for each sample, and the mean was calculated.

### Scanning electron microscopy

The scanning electron microscopy (SEM) analysis of the sea bass muscle was performed according to the method described by Liu et al. (34). The fillet was cut into 5 mm × 5 mm × 2 mm pieces, fixed with 2.5% glutaraldehyde solution at 4°C for 24 h, then rinsed with 0.1 M phosphate buffer (pH = 7.2) for 15 min, repeated three times. The washed sample was gradient dehydration with ethanol (50, 70, 80, 90, and 100%). Thereinto, the sample was dipped in anhydrous ethanol for 20 min twice, for 15 min in others. After freeze-drying and coating with gold, the specimen was observed using a Hitachi S-3400N scanning electron microscope (Hitachi S-3400N, Hitachi, Ltd., Tokyo, Japan).

### Extraction of myofibrillar protein

Myofibrillar protein (MP) was extracted from sea bass using the method described by Ding et al. (35). Briefly, the white muscle from the fillet was minced and rinsed with four times the weight of low-salt buffer (0.05 M NaCl, 0.02 M Tris–HCl, pH = 7.5) and homogenized using a homogenizer (NANOJ H10, ATS Engineering Inc., Germany) for 2 min. The homogenate was centrifuged at 5,000 × *g* at 4°C for 10 min. After separating the supernatant containing the sarcoplasmic proteins, the precipitate was rinsed twice using the low-salt buffer. Afterward, the obtained precipitate was extracted at 4°C for 20 h with four times the weight of high-salt buffer (0.45 M NaCl, 0.02 M Tris–HCl, pH = 7.5). After centrifugation (12,000 × *g*, 15 min, 4°C), the supernatant was poured into ten times the weight of precool deionized water and the mixture was incubated for 30 min at 4°C to precipitate MP. Finally, the resulting precipitate (MP) was collected by centrifugation (12,000 × *g*, 10 min, 4°C), and dissolved with 0.6 M NaCl, 0.02 M Tris–HCl buffer (pH = 7.5) to prepare MP mother solution. The concentration of MP was determined by the BCA method employing bovine serum albumin as the standard.

### Determination of total sulfhydryl content and Ca^2+^-ATPase activity

A total SH measurement kit (Nanjing Jiancheng Bioengineering Institute, China) was used to measure the total SH content of MP according to its instruction. The MP solution was diluted to 2.5–4 mg/ml with 0.6 M NaCl (pH = 7.0). In order to denature the protein, the diluted MP solution (0.5 ml) was added to 4.5 ml of 0.2 M Tris–HCl buffer (pH = 6.8) containing 8 M urea, 2.0% SDS and 0.01 M EDTA. To determine the total SH content, 0.4 ml of 0.1% 5, 5-dithiobis (2-nitrobenzoic acid) (DTNB) in 0.2 M Tris–HCl buffer (pH = 8.0) was added to 4 ml of the mixture containing denatured proteins and incubated at 40°C for 25 min. The absorbance of the reaction mixture at 412 nm was determined using an UV-5200 spectrophotometer (Shanghai Metash Instruments Co., Ltd, Shanghai, China), and SH concentration was calculated by the Equation 3. The results of total SH content were expressed as μmol/g prot.


(3) SH⁢concentration⁢(μm⁢o⁢l/g⁢p⁢r⁢o⁢t)⁢=A×D/(B×C)

where *A* is the measured absorbance, *B* is the concentration of MP solution, *C* is the molar extinction coefficient of 13,600 M^–1^ cm^–1^, and *D* is the dilution volume.

A Ca^2+^-ATPase kit (Nanjing Jiancheng Bioengineering Institute, China) was applied to determine the Ca^2+^-ATPase activity of MP according to its instruction. An aliquot (1 ml) of the diluted MP solution (2.5–8 mg/ml) was added to 0.6 ml of 0.5 M Tris-maleate buffer (pH = 7.0), and 1 ml of 0.1 M calcium chloride. The total volume of the reaction solution was supplemented with deionized waster to 9.5 ml. The reaction was initiated by the addition of 0.5 ml of ATP (0.02 M). After incubation at 25°C for 8 min, the reaction was terminated by adding 5 ml of 15% (w/v) trichloroacetic acid (TCA), and the reaction mixture was centrifuged at 3,500 × *g* for 5 min. The blank was carried out by adding 15% TCA before the diluted MP solution (2.5–8 mg/ml) was added. The inorganic phosphate liberated in the supernatant was measured by the method of Benjakul et al. (36). The results of Ca^2+^-ATPase activity were expressed by the concentration of released inorganic phosphate (Pi) indicated as μmol Pi/mg prot/h.

### Circular dichroism spectroscopy

The secondary structure of MP was investigated by a circular dichroism (CD) spectropolarimeter (Jasco J-1500-150, Jasco Corp., Tokyo, Japan). The MP solution was put into a dialysis bag and dialyzed for 12 h at 4°C. A 300 μl aliquot of the dialyzed MP solution (0.2 mg/ml) was placed in a 1 mm quartz CD cell (Hellma, Muellheim, Baden, Germany). The main parameters were set as follows: 1.0 nm bandwidth, 1 s D.I.T., 200 mdeg/1.0 dOD of CD and FL scale, 50 nm/min scan speed and 0.1 nm/data step resolution. The spectral scanning range was from 190 to 260 nm. Three scans were averaged to obtain one spectrum. Circular dichroism of MP structure was expressed by the mean specific ellipticity [θ] (deg⋅cm^2^⋅dmo^*l*–1^). The percentage of the secondary structure of MP was evaluated by Yang et al. (37) method.

### Statistical analysis

Statistical data were subjected to analysis of one-way ANOVA followed by the Duncan procedure between means (significance was defined at *p* < 0.05) using IBM SPSS Statistics 25 (SPSS Inc., Chicago, IL, USA). Unless otherwise stated, all results were presented as the means ± standard deviation (SD). The figures were drawn by Origin 2019b (Origin Lab, Northampton, MA, USA).

## Results and discussion

### Effect of static magnetic field treatment on total viable counts

**FIGURE 1:**
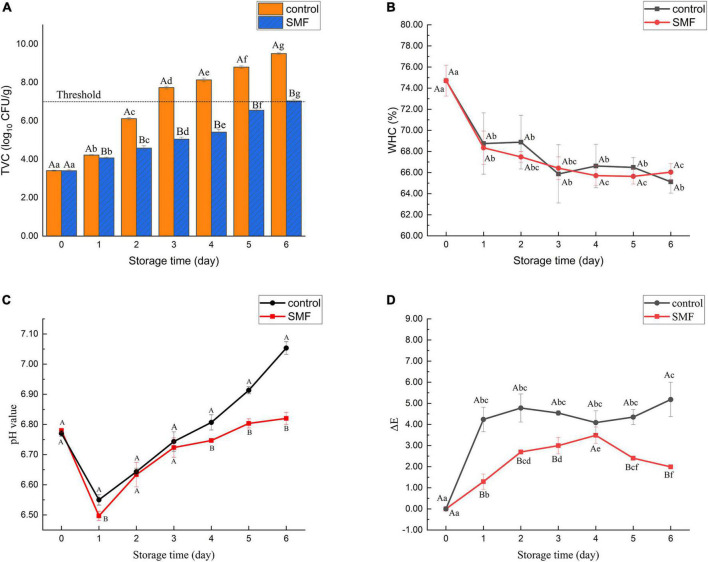
Changes in TVC **(A)**, WHC **(B)**, pH value **(C)**, and color differences **(D)** of sea bass fillets with different treatments. The dotted line in panel **(A)** shows the upper acceptable limit (7.0 log_10_ CFU/g). SMF: treated with static magnetic field (5 mT) at 4°C, control: storage at 4°C without SMF. Different capital letters indicate statistically significant differences between samples with different treatments on the same day (*p* < 0.05), and different lowercase letters indicate statistically significant differences between samples with different storage time under the same treatment (*p* < 0.05), the same below.

Changes in TVC of the sea bass fillet with different treatments are shown in Figure 1A. The initial count in the sample was 3.4 log_10_ CFU/g, indicating excellent quality of fresh fish. This load was similar to that of Japanese sea bass (*Lateolabrax japonicus*) reported by Li et al. (2). After 3 days of storage, it was observed that TVC of the control exceeded the maximum acceptable level of TVC (7.0 log_10_ CFU/g) of freshwater and marine fish (38). By contrast, microbial counts in sea bass fillets treated with SMF for 6 days exceeded the edible limits. Therefore, these results reveal that SMF treatment could effectively extend the shelf life of the sea bass fillet from a microbiological point of view. Lins et al. (39) reported that 1 Hz pulsed magnetic field treatment (PMF, 10 mT), a type of time-varying magnetic field, for 2 h could reduce microbial counts in fresh beef during cold storage, while continuing exposure to PMF for 12 days did not significantly inhibit the growth of bacteria. They suggested that the surviving bacteria might have adapted to the PMF. In the present study, similar or even better bacteriostatic effects could be achieved by SMF treatment of 5 mT, while the energy required was lower.

Previous studies have suggested that the antimicrobial effect of SMF may rely on alterations in membrane calcium ion flux, which would result in the deformation of imbedded ion channels, thereby altering their activation kinetics and influencing several biological systems (40, 41). In addition, the inactivation efficacy of microorganisms depends on magnetic field process parameters (such as type, intensity, frequency, and duration of action), microorganism properties (such as types, growth phase, and density) (28). Consequently, the window effect hypothesis has been proposed for the different effects of magnetic field on the growth of microorganisms, that is, specific parameters correspond to specific microorganisms (42, 43).

### Effect of static magnetic field treatment on water holding capacity

Water holding capacity is a crucial attribute reflecting fish quality, and it may represent the ability of muscle protein to prevent water from being released under external forces. As shown in Figure 1B, a decrease in WHC was observed in the sea bass fillet treated with and without SMF, and no significant difference was observed between them during the entire cold storage. Some studies confirmed that the WHC was often related to protein structures (44, 45). During storage, the denaturation of myosin will alter the water distribution in the fish, resulting in a considerable loss of the free water content in fish and a significant decrease in the WHC. Therefore, our results suggest that significant protein denaturation may occur in sea bass fillets. Yang et al. (46) found that the direct current magnetic field (DC-MF) treatment could improve WHC of MP gels, and this might be attributed to protein unfolding, re-crosslinking and aggregation induced by DC-MF. The results of the present study are inconsistent with these. It is possible that, due to the differences in biological characteristics between raw materials, differences in WHC of sea bass fillets are more difficult to detect during exposure to SMF.

### Effect of static magnetic field treatment on pH

Figure 1C shows the changes of pH in the sea bass fillet with different treatments. The pH of all samples decreased in the early stage of storage and then increased gradually, while the pH of the SMF-treated samples remained consistently lower than that of the control (*p* < 0.05). One possible explanation for the initial decrease in pH could be the accumulation of lactic acid, a product of glycolysis (47). In glycolysis, glucose is metabolized to the final product pyruvate, which is converted to lactic acid by lactate dehydrogenase (48). However, there are no published data showing that lactate dehydrogenase activity is directly modulated by SMF, so further exploration is required. In addition, the accumulation of alkaline substances such as amines and ammonia produced by spoilage bacteria may be responsible for the increase of pH value of fish samples in the late period of storage (28, 49, 50). Thus, this result is consistent with the change of microbial counts stated in section “Effect of static magnetic field treatment on total viable counts,” and demonstrate that SMF treatment not only inhibits the growth of microorganisms but also restrains other autolytic processes that generate alkaline substances.

### Effect of static magnetic field treatment on color

Color is one of the most direct and satisfactory indicators for consumers to evaluate the freshness of fish (51). Figure 1D shows changes in absolute color difference (ΔE) of the sea bass fillet with different treatments. The values of ΔE presented an increasing trend during storage, and the ΔE value of the control sample was higher than that in the SMF-treated sample (*p* < 0.05). Color differences larger than 2–4 are considered perceptible to consumers (52). In our data, the sea bass fillet treated with SMF would be perceived as less color change than the control fillet by consumers, indicating that SMF treatment could delay the discoloration of fish fillets. As recorded in Table 1, the increase in ΔE value may be due to changes in a* and b* values, since changes in L* value of the SMF-treated samples were similar to those of the control. Hence the color variation between samples is mainly reflected in redness and yellowness, and the reasons for these changes are discussed below. In addition, the changes in the refractive index of the fish surface caused by decreased WHC could directly affect the L* value of fish (21). As discussed in section “Effect of static magnetic field treatment on water holding capacity,” SMF treatment did not cause significant enhancement in WHC, which was reflected in the similar changes in L* value between the SMF-treated and control samples.

**TABLE 1:** Effect of static magnetic field on color of the sea bass fillet during cold storage at 4°C.

	L*		a*		b*	
Storage time (day)	Control	SMF	Control	SMF	Control	SMF
0	44.19 ± 1.05^a^	41.11 ± 0.95^a^	−0.66 ± 0.47^a^	−1.69 ± 0.09^a^	−3.24 ± 0.18^a^	−4.16 ± 0.27^a^
1	39.99 ± 0.50^bc^	40.21 ± 0.79^ab^	−0.45 ± 0.41^ab^	−0.96 ± 0.28^b^	−2.9 ± 0.72^a^	−3.62 ± 0.62^ab^
2	39.53 ± 1.81^b^	38.80 ± 1.33^b^	−0.09 ± 0.41^b^	−0.78 ± 0.34^bc^	−2.54 ± 0.74^ab^	−3.33 ± 0.90^ab^
3	40.11 ± 1.12^bc^	39.25 ± 0.91^b^	0.66 ± 0.46^c^	0.27 ± 0.41^d^	−1.82 ± 0.74^bc^	−2.89 ± 0.63^b^
4	40.68 ± 0.92^bc^	38.20 ± 1.02^b^	−0.66 ± 0.40^a^	−0.28 ± 0.79^c^	−1.29 ± 1.10^c^	−3.03 ± 0.79^b^
5	41.03 ± 1.09^c^	38.93 ± 1.09^b^	−0.73 ± 0.28^a^	−0.86 ± 0.40^b^	−0.06 ± 0.74^d^	−3.67 ± 0.44^ab^
6	42.49 ± 0.55^d^	39.59 ± 1.10^ab^	−0.78 ± 0.46^a^	−0.72 ± 0.38^bc^	1.65 ± 0.86^e^	−3.36 ± 0.33^ab^

Results are presented as the mean ± SD. Different lowercase letters in a column indicate statistically significant differences between samples with different storage time (*p* < 0.05). SMF: treated with static magnetic field (5 mT) at 4°C, control: storage at 4°C without SMF.

The a* value of meat is determined by the rate of oxymyoglobin oxidation and metmyoglobin reducing activity (53, 54). Oxymyoglobin is redness in color and is produced when myoglobin is oxygenated or exposed to oxygen, while methemoglobin is brown in color and occurs when oxygen concentration is between 0.5 and 1% or when meat is exposed to air for a long time (55). The initial a* value for SMF-treated fillet was lower than that in the control at day 0, which may be due to the inherent color differences between individuals. In the early stage of storage, the a* value of the fillet treated with SMF increased more, indicating that the myoglobin in the fillet was accelerated to form oxymyoglobin, which may be related to the fact that magnetic field treatment could promote the unfolding of myoglobin structure (56). Myoglobin would be more susceptible to oxidative attack due to the unfolded structure. In the later stage of storage, decreased a* values were observed in both SMF-treated and control fillets, while the decrease trend was relatively slighter in the fillets treated with SMF. The similar tendency of a* value for the SMF-treated fish was also found for other refrigerated meat products, such as static magnetic field extended supercooling (SM-ES) treated beef (25). This is not surprising since longer-stored fish fillets were expected to contain high levels of methemoglobin, which cause the surface color of the fillet to shift toward brown. Previous results in the literature have proven that low frequency magnetic field treatments (3, 6, 9, 12 mT, 50 Hz) for 10 h could effectively inhibit the increase of methemoglobin content in the solution of horse skeletal muscle myoglobin (57). Therefore, these results confirm that SMF treatment could stabilize the redness of the fish fillet.

The b* values in all sea bass fillets showed an increasing tendency during cold storage, expressing an evolution toward gray yellow tones as the fillets aged. Our results are consistent with data reported by Cai et al. (58), showing a significant increase in the b* parameter of Japanese sea bass fillets stored under refrigerated conditions. On the other hand, the b* value of the sea bass fillet increased more slowly when treated with SMF, suggesting the yellowish of SMF-treated fillets was not pronounced. Yellow color is often associated with lipid and protein oxidation (59). Therefore, the significant decrease detected in the b* value of the SMF-treated fillet might be attributed to SMF-induced antioxidant effects.

### Effect of static magnetic field treatment on textural properties

Textural properties are valued quality indicators reflecting sensory and functional properties of fish. The variations in texture parameters of the sea bass fillet with different treatments are presented in Table 2. The TPA results showed that after 6 days of storage, the hardness, springiness, cohesiveness, and chewiness of the control sample decreased by 69.5, 24.6, 19.0, and 86.7%, respectively. These results indicated that the fish fillet began to soften and lose its elasticity. However, the corresponding values for the SMF-treated sample decreased by 50.5, 29.3, 4.8, and 76.3%, respectively. The evaluation results of texture properties showed that the SMF-treated sea bass fillet had a better texture than the control. A similar phenomenon was reported by Zhu et al. (31), who found that alternating magnetic field treatment (AMF, 5 mT), a type of time-varying magnetic field, could further delay the texture deterioration of ultra-high pressure treated shrimp, while AMF treatment had an adverse effect on the color of shrimp.

**TABLE 2:** Effect of static magnetic field on texture of the sea bass fillet during cold storage at 4°C.

	Hardness (g)		Springiness		Cohesiveness		Chewiness	
Storage time (day)	Control	SMF	Control	SMF	Control	SMF	Control	SMF
0	2,304.0 ± 143.3^Aa^	2,288.0 ± 124.3^Aa^	0.57 ± 0.06^Aa^	0.58 ± 0.05^Aa^	0.42 ± 0.04^Aa^	0.42 ± 0.04^Aa^	786.8 ± 133.2^Aa^	786.8 ± 175.2^Aa^
1	1,796.8 ± 113.5^Ab^	1,968.1 ± 91.4^Ab^	0.48 ± 0.03^Ab^	0.49 ± 0.03^Abc^	0.41 ± 0.04^Aa^	0.40 ± 0.04^Aa^	350.7 ± 31.9^Ab^	382.3 ± 54.0^Ab^
2	1,417.3 ± 141.0^Ac^	1,641.0 ± 163.6^Ac^	0.43 ± 0.04^Ab^	0.46 ± 0.02^Abc^	0.38 ± 0.03^Aa^	0.38 ± 0.02^Aa^	225.0 ± 28.2^Ac^	291.5 ± 51.8^Abc^
3	1,386.8 ± 79.0^Bc^	1,568.5 ± 32.6^Acd^	0.46 ± 0.01^Ab^	0.46 ± 0.03^Abc^	0.39 ± 0.02^Aa^	0.38 ± 0.02^Aa^	248.1 ± 18.6^Abc^	276.2 ± 24.2^Abc^
4	1,249.7 ± 121.2^Acd^	1,355.5 ± 56.1^Ade^	0.46 ± 0.04^Ab^	0.46 ± 0.01^Abc^	0.38 ± 0.02^Aa^	0.39 ± 0.01^Aa^	218.8 ± 45.0^Ac^	260.1 ± 24.0^Abc^
5	1,135.9 ± 157.4^Ad^	1,191.5 ± 119.6^Aef^	0.42 ± 0.03^Ab^	0.45 ± 0.03^Abc^	0.39 ± 0.03^Aa^	0.40 ± 0.02^Aa^	189.8 ± 35.9^Acd^	216.0 ± 29.6^Ac^
6	702.0 ± 66.9^Be^	1,132.4 ± 96.42^Af^	0.43 ± 0.02^Ab^	0.41 ± 0.01^Ac^	0.34 ± 0.07^Aa^	0.40 ± 0.03^Aa^	104.5 ± 29.9^Bd^	186.6 ± 20.6^Ac^

Results are presented as the mean ± SD. Different capital letters in a row indicate statistically significant differences between samples with different treatments (*p* < 0.05); different lowercase letters in a column indicate statistically significant differences between samples with different storage time (*p* < 0.05). SMF: treated with static magnetic field (5 mT) at 4°C, control: storage at 4°C without SMF.

Muscle texture in fish is determined by several intrinsic biological parameters, such as the density of muscle fiber and the content of fat and collagen (32). Besides, autolysis is triggered by the death of the fish, softening the muscles under the combined effect of microbial activity (60). In our study, it was observed that microbial activity was inhibited by SMF treatment, which resulted in lower bacterial load and slighter protein degradation in the SMF-treated fillet. Therefore, the SMF-treated fillet exhibited significant improvements in textural properties, probably due to the magnetic field-induced inactivation of microorganisms and changes in the autolysis process.

### Effect of static magnetic field treatment on total sulfhydryl content of myofibrillar protein

**FIGURE 2:**
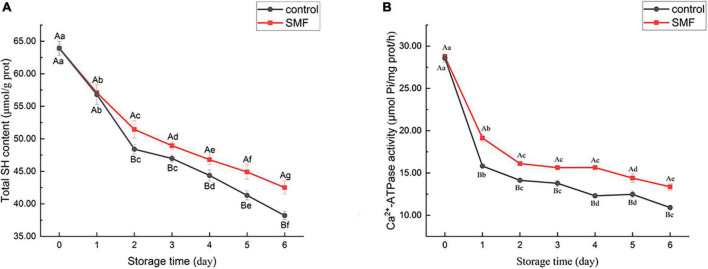
Changes in total SH content **(A)** and Ca^2+^-ATPase activity **(B)** of sea bass fillets with different treatments.

Sulfhydryl groups are present in MP, mainly in the head of myosin (61). The content of SH groups is recognized as an important indicator of the integrity of myosin (36). Changes in the total SH content of MP extracted from sea bass muscles with different treatments are shown in Figure 2A. Compared with fresh muscle, the final content of total SH in SMF-treated and control samples were 42.52 and 38.21 μmol/g prot, which decreased by 33.5 and 40.2%, respectively. The reduction in SH group content has been reported to be due to the formation of disulfide bonds through oxidation of SH groups or disulfide interchanges (62). As can be seen, SMF-treated samples tended to show less decrease in total SH content, particularly in later stage of storage. In other words, SMF treatment could suppress the oxidation of SH groups in MP during cold storage. Protein oxidation would modify the amino acid sidechains and alter the protein polypeptide backbone, resulting in structural changes in the protein (63, 64). The ion–protein dissociation proposed by Bingi and Savin (65) might explain the effect of magnetic field on protein oxidation, when an ion enters a protein cavity containing a ligand, it would undergo directional movement under the influence of the magnetic field. Such directional movement may induce the formation of local micro-electric fields, which disturb the original equilibrium distribution in the ion cloud, thereby causing different biological responses (16).

### Effect of static magnetic field treatment on Ca^2+^-ATPase activity of myofibrillar protein

The Ca^2+^-ATPase activity is an important indicator of the integrity of myosin. Any change in the conformation of myosin would lead to a decrease in enzyme activity (66, 67). A notable decrease in Ca^2+^-ATPase activity was observed in all samples (Figure 2B), indicating that myosin underwent denaturation. Thus, the denaturation of myofibrillar proteins during cold storage may account for the reduction in WHC. Myofibrillar proteins, which are primarily responsible for water-binding properties, suffered severe denaturation and, consequently, significantly reduced WHC was observed in both control and SMF samples.

It is noteworthy that the decrease of Ca^2+^-ATPase activity was remarkably lower in the presence of SMF than in their absence (*p* < 0.05). Loss of Ca^2+^-ATPase activity is postulated to be the conformational changes and aggregation in the globular head of myosin (61, 68). Moreover, the loss of Ca^2+^-ATPase activity could also be caused by the oxidation of SH_1_ and SH_2_ on the active site of actomyosin (36, 69). In our study, the decrease in Ca^2+^-ATPase activity was in agreement with the decrease in total SH content (Figure 2A). In addition, protein rearrangements *via* protein–protein interactions are also considered to be responsible for the loss in Ca^2+^-ATPase activity (70). Overall, the above researches demonstrated that the loss in Ca^2+^-ATPase activity is associated with the denaturation and oxidation of protein, and alteration in protein conformation, and our results suggest that SMF treatments could effectively retard these processes and stabilize the structure of protein.

### Effect of static magnetic field on muscle microstructure

**FIGURE 3:**
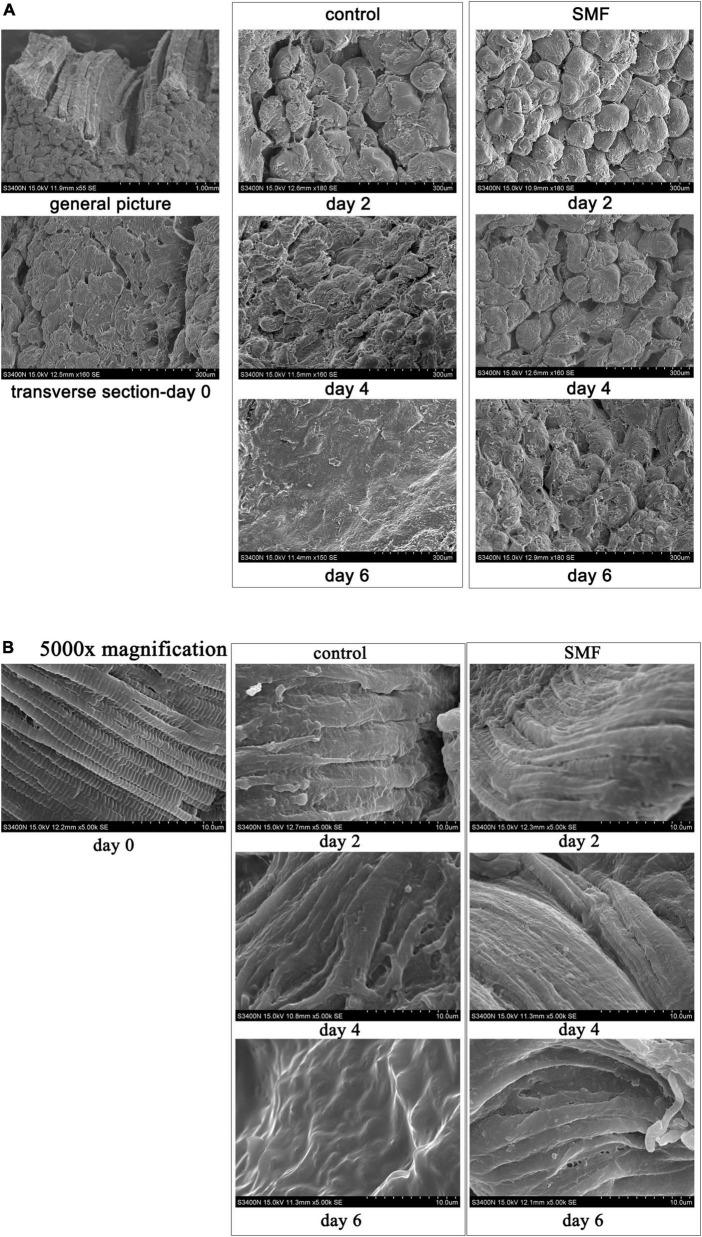
Microstructure of the sea bass muscle observed by SEM. **(A)** Transverse sections, **(B)** transverse sections at 5,000× magnification.

The microstructural changes in the transverse direction of the sea bass muscle with different treatments are shown in Figure 3. At day 0, single myofiber could be easily distinguished in the transverse fiber fracture, and the boundary between the myofibers was clear (Figure 3A). From the micrographs of control samples, clear changes in the cross-sectional structure of myofibers were visible. The boundaries between the adjacent myofibers were not visible any longer, and myofibers had largely agglomerated into lamellae. Compared with the controls, SMF treatment could reduce the structural changes of myofibers in the sea bass muscle during storage. No significant change was apparent in the myofibers, and the boundary between the myofibers was clearly identified, under SMF treatment of 5 mT for 2 and 4 days, respectively. After 6 days of SMF treatment, distorted myofibers were also observed, but to a lesser extent, suggesting a slower development of the structural changes under SMF treatment.

In addition, higher magnification of sea bass muscle was used to examine the myofiber structure in more detail. The sarcomere is the basic contractile unit of the striated muscle and is arranged in a stacked fashion throughout the muscle tissue (71). The defined brick structure of the sarcomere, intact and compact, was obviously observed in the fresh sample at day 0 (Figure 3B). After 6 days of storage at 4°C, it was not possible to identify the individual sarcomere or even transverse elements, and the striated structure of the myofibers disappeared and the contractile elements of the muscle fibers disintegrated. Applying SMF of 5 mT stabilized the structure of sarcomere, where Z-band (the dividing line between the sarcomeres) was visible (day 2), although the sarcomere structure appeared to be disrupted in samples treated with SMF for 6 days. These might suggest that SMF treatment partly protects sea bass muscle fibers from damage caused by aging and microbial associated proteolysis, as shown in sea bass (72).

### Effect of static magnetic field treatment on the secondary structure of myofibrillar protein

**FIGURE 4:**
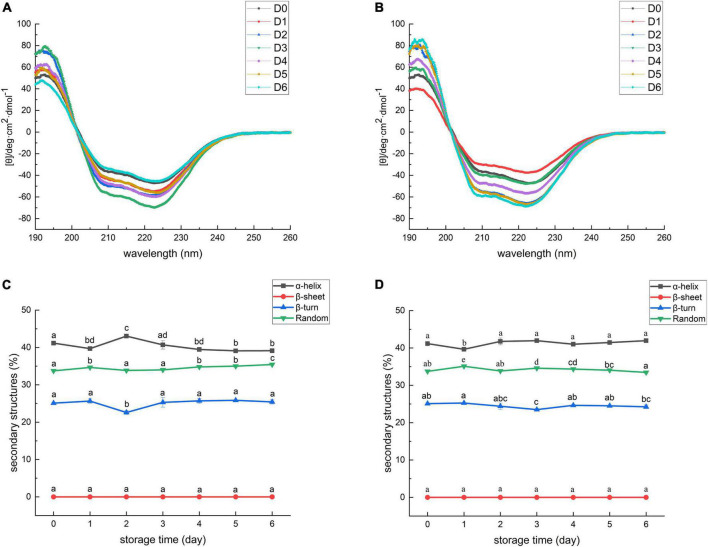
Circular dichroism spectra of myofibrillar protein from sea bass. **(A)** Control; **(B)** SMF. D0, 1, 2, 3, 4, 5, 6 represent day 0, 1, 2, 3, 4, 5, 6, respectively. Secondary structure of myofibrillar protein from sea bass. **(C)** Control; **(D)** SMF. Different lowercase letters on the top of bars indicated statistically significant differences between samples (*p* < 0.05).

The CD spectrums of MP extracted from sea bass muscles with different treatments are shown in Figures 4A,B. The CD spectrum of all samples exhibited two main minima at around 208 and 222 nm. The two bands were rationalized by the *n*-π* transition in the peptide bond of α-helix, therefore, corresponding to the changes in α-helical structure (73). Secondary structure analysis of MP is exhibited in Figures 4C,D. The percentages of α-helix, β-turn, and random coil in SMF-treated samples had no significant decrease from day 0 to day 6 (*p* > 0.05), implying that the secondary structure of MP did not undergo severe damage. Conversely, the percentages of α-helix in the controls decreased whilst the percentages of random coil increased, suggesting a gradual loss of the helical structure in MP. The α-helix component was likely transformed into the random coil. Similar results were also shown in a study reported by Zhang et al. (74), in which the secondary structure of grouper (*Epinephelus coioides*) protein transformed from α-helix to random coil during cold storage. These results suggest that SMF treatment was effective in preserving the α-helix structure.

The gradual deletion of α-helix in MP was related to structural changes in myosin, including the unfolding of the α-helix structure of the myosin rod portion (75). As described in section “Effect of static magnetic field treatment on total sulfhydryl content of myofibrillar protein” and “Effect of static magnetic field treatment on Ca^2+^-ATPase activity of myofibrillar protein,” myosin was subjected to severe oxidation and denaturation during cold storage and, consequently, the decrease in the content of α-helix was detected. In addition, the content of α-helical structure is also related to the hydrogen bond stability between the carbonyl oxygen and amino hydrogen of the polypeptide chain (34). Enhanced strength of hydrogen bonds after magnetic field treatment has been previously reported in some bacteria and cells (76, 77). She et al. (77) described the transition of random coils to α-helices, and intermolecular β-sheets to intramolecular ones in the secondary structure of proteins in *E. coli* after SMF treatment at 10 T for 30 min, and they suggested that this may be due to the destruction of intermolecular cohesion and the enhancement of intramolecular hydrogen bonds. Thus, differences in the stability of α-helical structures between SMF-treated and control samples could be attributed to SMF-induced effects, including antioxidant effects and enhancement of intramolecular hydrogen bonds.

## Conclusion

This study investigated the feasible application of SMF treatment (5 mT) in mitigating the quality deterioration of the sea bass fillet during cold storage. The results of microbiological and physicochemical properties showed that SMF treatment could effectively slow down the rate of quality deterioration of the sea bass fillet. SMF treatment not only inhibited microbial growth, but also suppressed the protein structural changes. The inhibition of microbial metabolism and the stabilization of protein structure under SMF treatment resulted in lower pH and ΔE values, and the better texture in SMF-treated samples. Therefore, our results show that SMF treatment could be utilized as an effective approach for maintaining the quality of fish. In this study, the beneficial effects of SMF treatment on maintaining the quality of sea bass fillets may be limited, which is associated with magnetic field parameters, whereas the mechanism of SMF-induced effects is also worth exploring. Thus, future research working on SMF-assisted fish preservation should include optimal intensity, operating time, distribution of SMF, as well as whether the responses of different fish species are consistent.

## Data availability statement

The original contributions presented in this study are included in the article/supplementary material, further inquiries can be directed to the corresponding author.

## Author contributions

LT: methodology, validation, investigation, data curation, formal analysis, visualization, and writing—original draft and review and editing. HT: supervision, resources, and writing—review. JC: investigation and writing—review and editing. SS: conceptualization. RL: data curation. ZZ: methodology. CO: conceptualization, supervision, project administration, resources, funding acquisition, and writing—review and editing. All authors contributed to the article and approved the submitted version.
